# Acute kidney injury in Turkey: epidemiological characteristics, etiology, clinical course, and prognosis

**DOI:** 10.1186/s12882-022-02933-1

**Published:** 2022-10-05

**Authors:** Meltem Gursu, Itir Yegenaga, Serhan Tuglular, Belda Dursun, Sibel Gokcay Bek, Simge Bardak, Engin Onan, Serap Demir, Ulver Derici, Ayhan Dogukan, Mustafa Sevinc, Ismail Kocyigit, Eda Altun, Ali Burak Haras, Mehmet Riza Altiparmak, Halil Zeki Tonbul

**Affiliations:** 1 Faculty of Medicine, Department of Internal Medicine, Division of Nephrology, Bezmialem Vakıf University, Istanbul, Turkey; 2 Faculty of Medicine, Department of Internal Medicine, Division of Nephrology, Maltepe University, Istanbul, Turkey; 3 Faculty of Medicine, Department of Internal Medicine, Division of Nephrology, Marmara University, Pendik Training and Research Hospital, Istanbul, Turkey; 4 Faculty of Medicine, Department of Internal Medicine, Division of Nephrology, Pamukkale University, Denizli, Turkey; 5 Faculty of Medicine, Department of Internal Medicine, Division of Nephrology, Kocaeli University, Kocaeli, Turkey; 6 Department of Internal Medicine, Division of Nephrology, Batman State Hospital, Batman, Turkey; 7 Faculty of Medicine, Department of Internal Medicine, Division of Nephrology, Cukurova University, Adana, Turkey; 8 Faculty of Medicine, Department of Internal Medicine, Division of Nephrology, Mersin University, Mersin, Turkey; 9 Faculty of Medicine, Department of Internal Medicine, Division of Nephrology, Gazi University, Ankara, Turkey; 10 Faculty of Medicine, Department of Internal Medicine, Division of Nephrology, Firat University, Elazig, Turkey; 11 Department of Internal Medicine, Division of Nephrology, University of Health Sciences, Sisli Hamidiye Etfal Training and Research Hospital, Istanbul, Turkey; 12 Faculty of Medicine, Department of Internal Medicine, Division of Nephrology, Erciyes University, Kayseri, Turkey; 13 Department of Internal Medicine, Division of Nephrology, Golcuk State Hospital, Kocaeli, Turkey; 14 Department of Internal Medicine, Division of Nephrology, University of Health Sciences, Dr. Lutfi Kirdar City Hospital, Istanbul, Turkey; 15 Cerrahpasa Faculty of Medicine, Department of Internal Medicine, Division of Nephrology, Istanbul University-Cerrahpasa, Istanbul, Turkey; 16 Faculty of Medicine, Department of Internal Medicine, Division of Nephrology, Meram University, Konya, Turkey

**Keywords:** Acute kidney injury, Etiology, Kidney replacement therapy, Survival

## Abstract

**Background:**

This study aimed to evaluate the etiologies, comorbidities, and outcomes of acute kidney injury (AKI) in Turkey and determine any potential differences among different geographical parts of the country.

**Methods:**

This prospective observational study was conducted by the Acute Kidney Injury Working Group of the Turkish Society of Nephrology. Demographical and clinical data of patients with AKI at the time of diagnosis and at the 1^st^ week and 1^st^, 3^rd^, and 6^th^ months of diagnosis were evaluated to determine patient and renal survival and factors associated with patient prognosis.

**Results:**

A total of 776 patients were included (54.7% male, median age: 67 years). Prerenal etiologies, including dehydration, heart failure, and sepsis, were more frequent than other etiologies. 58.9% of the patients had at least one renal etiology, with nephrotoxic agent exposure as the most common etiology. The etiologic factors were mostly similar throughout the country. 33.6% of the patients needed kidney replacement therapy. At the 6^th^ month of diagnosis, 29.5% of the patients had complete recovery; 34.1% had partial recovery; 9.5% developed end-stage kidney disease; and 24.1% died. The mortality rate was higher in the patients from the Eastern Anatolian region; those admitted to the intensive care unit; those with prerenal, renal, and postrenal etiologies together, stage 3 AKI, sepsis, cirrhosis, heart failure, and malignancy; those who need kidney replacement therapy; and those without chronic kidney disease than in the other patients.

**Conclusion:**

Physicians managing patients with AKI should be alert against dehydration, heart failure, sepsis, and nephrotoxic agent exposure. Understanding the characteristics and outcomes of patients with AKI in their countries would help prevent AKI and improve treatment strategies.

## Background

Acute kidney injury (AKI) is an important part of nephrology practice in both inpatient and outpatient clinics as well as emergency departments. Different criteria have been suggested for the diagnosis of AKI during the last decade. AKI has been most recently described by the Kidney Disease: Improving Global Outcomes (KDIGO) in 2012 [1].

The incidence of AKI has been reported to vary among different studies, possibly in part owing to the changing criteria for the diagnosis and diversity of the study populations. AKI is a multifactorial condition; many clinical conditions and medications contribute to its development. However, more importantly, AKI has been found to be associated with increased morbidity and mortality rates and worst outcomes, regardless of the etiology [2]. Both the etiology and prognosis of AKI may differ among regions with different socioeconomic statuses. The presence of AKI is associated not only with short-term prognosis but also with long-term mortality. Lafrance et al. [2] retrospectively analyzed 82711 hospitalized US veteran patients with AKI. Approximately 17.4% of patients who survived at least 90 days after AKI onset were reported to die during follow-up, and the mortality rate was higher in those with more severe AKI than in their counterparts. Besides patient mortality, AKI was found to be associated with worse renal survival and may lead to chronic kidney disease (CKD). Iram et al. [3] reported that 4.35% of their patients with AKI died, and 28.69% developed CKD. Similar reports can be found in the literature [4–6].

This multicenter study aimed to evaluate the etiologies, clinical courses, treatment options, and outcomes of AKI in Turkey and determine any potential differences among different geographical regions of the country.

## Methods

This prospective observational study was conducted by the Acute Kidney Injury Working Group of the Turkish Society of Nephrology. It was announced online to all members of the Turkish Society of Nephrology, and only the respondents were recorded as the investigators. The investigators were asked to evaluate their patients with AKI at inpatient and emergency clinics as well as intensive care units (ICUs) according to the criteria proposed by the KDIGO: sudden decline in kidney function (increase in the serum creatinine level of > 0.3 mg/dL within 48 h, or > 50% within 7 days, or decreased urine output of < 0.5 mL/kg/h for more than 6 h) [1]. All parameters of the patients with AKI who provided informed consent were recorded into a web-based database. The study was conducted between April 2018 and 2019.

The demographical parameters, including age, sex, date and place of AKI diagnosis, the clinic where the patient was diagnosed to have AKI, status of hospitalization, duration between initial hospitalization and AKI diagnosis, and basal creatinine level and estimated glomerular filtration rate (eGFR), were recorded for each patient. The serum creatinine level assessed before 48 h was considered the basal creatinine level during hospital follow-up, while and in patients with AKI at the time of admission the creatinine level at the previous week, when available, was considered the basal level. The stage of AKI as defined by the KDIGO was recorded.

The etiology of AKI was recorded and classified into three major subgroups: prerenal, renal, and postrenal. Prerenal etiologies included dehydration, gastrointestinal losses, heart failure, burns, sepsis, liver cirrhosis, and ascites. Renal factors included ischemic events, nephrotoxin exposure, glomerulonephritis, vasculitis, tubulointerstitial nephritis, and diabetic nephropathy. Nephrotoxic agents included aminoglycosides, radiocontrast material, non-steroidal anti-inflammatory drugs (NSAIDs), renin–angiotensin–aldosterone system (RAS) blockers, colistin, amphotericin, chemotherapeutic agents, and other drugs. Postrenal factors included stone and malignancies of the urinary tract, prostatic disease, retroperitoneal fibrosis, and vesicoureteral reflux. The clinicians were free to select more than one option.

The clinicians were asked to select one or more causes of hospitalization, including respiratory, cardiovascular, cerebrovascular, hematological, gastroenterological, hepatological, and genitourinary diseases; infections; immunosuppressive status; cardiovascular surgery; and major or minor non-cardiac surgery. Comorbidities included diabetes mellitus (DM), hypertension, ischemic cardiovascular disease, CKD, chronic obstructive pulmonary disease, heart failure, liver cirrhosis, and malignancies; they were described on the basis of the medical history of the patients.

The clinicians were asked to fill data regarding sepsis and systemic inflammatory response syndrome (SIRS). SIRS was diagnosed on the basis of the following findings: body temperature over 38 °C or below 36 °C, heart rate over 90 beats/min, respiratory rate over 20 breaths/min, PaCO_2_ level above 32 mmHg, white blood cell count more than 12000/mm^3^ or below 4000/mm^3^, or ratio of band forms over 10%. Sepsis was diagnosed when there was additional evidence of infection and organ dysfunction [7, 8].

Besides the initial evaluation, follow-up visits were performed at the 1^st^ week and 1^st^, 3^rd^, and 6^th^ months of the diagnosis of AKI. The clinicians recorded the outcome as complete recovery, partial recovery, end-stage kidney disease (ESKD) development, and mortality, as well as data related to kidney replacement therapy (KRT) at each visit, when needed. Complete recovery was defined as creatinine levels falling to the basal level, while partial recovery was defined as a > 50% decrease in the creatinine levels that did not reach the basal level. The types of KRT, including intermittent hemodialysis (IHD), continuous kidney replacement therapy (CKRT), and peritoneal dialysis (PD), were recorded. More than one option could be selected when used during follow-up. The demographical and clinical parameters associated with the prognosis of AKI were evaluated using a Cox regression analysis.

### Statistical analysis

The descriptive parameters were expressed as numbers and percentages for the categorical variables and medians and interquartile ranges (IQRs) for the numerical variables. The numerical variables of two independent groups were compared using the Mann–Whitney U test, as they were unevenly distributed. Meanwhile, the categorical variables were compared using the chi square test. The risk factors of mortality and ESKD in the patients with AKI were analyzed using a Cox regression analysis. The statistical alpha significance level was set at *p* < 0.05.

## Results

A total of 776 patients were analyzed after excluding those with missing data. Of them, 420 (54.1%) were men, and all were Caucasian, except for one black patient. The median patient age was 67 (IQR: 56–77) years. The centers providing data were classified according to the region of the country: Marmara-1, Marmara-2, and Central, Southern, and Eastern Anatolian regions. The names of the regions and centers and number of patients included according to region are presented in Table 1.

**Table 1:** The names of the regions and centers and number of patients included according to region

**Region**	**Center**	**n (%)**	**Population in 2019** [11]
Marmara-1 region	Bezmialem Vakif University, Faculty of Medicine, Istanbul Istanbul University-Cerrahpasa, Cerrahpasa Faculty of Medicine, Istanbul University of Health Sciences, Dr. Lutfi Kirdar City Hospital, Istanbul Maltepe University, Faculty of Medicine, Istanbul Marmara University, Faculty of Medicine, Pendik Training and Research Hospital, Istanbul University of Health Sciences, Sisli Hamidiye Etfal Training and Research Hospital, Istanbul	251 (32.3)	14,921,827
Marmara-2 region	Golcuk State Hospital, Kocaeli Kocaeli University, Faculty of Medicine, Kocaeli	106 (13.7)	1,940,551
Central Anatolian region	Erciyes University, Faculty of Medicine, Kayseri Gazi University, Faculty of Medicine, Ankara Meram University, Faculty of Medicine, Konya	83 (10.7)	9,062,835
Southern Anatolian region	Çukurova University, Faculty of Medicine, Adana Mersin University, Faculty of Medicine, Mersin Pamukkale University, Faculty of Medicine, Denizli	229 (29.5)	5,058,555
Eastern Anatolian region	Batman Regional State Hospital, Batman Fırat University, Faculty of Medicine, Elazig	107 (13.8)	1,195,197

AKI was diagnosed in 357 (47.9%) inpatients, 126 (16.9%) ICU patients, and 262 (35.2%) emergency clinic patients. The median duration between hospitalization and AKI diagnosis was 0 (IQR: 0–1) days. The median basal creatinine level and eGFR of the patients, excluding 153 patients with no data, were 1.20 (IQR: 0.89–1.80) mg/dL and 47 (IQR: 25–80) mL/min/1.73 m^2^, respectively.

The etiology of AKI is presented in Table 2. Prerenal etiologies were more frequent, with dehydration being the most prevalent, followed by heart failure and sepsis. 58.9% of the patients had at least one renal etiology, among which exposure to a nephrotoxic agent was the most frequent. NSAIDs, RAS blockers, and radiocontrast agents were the three most common nephrotoxic agents. Postrenal etiologies were recorded in 13.5% of the patients. A total of 275 (35.4%) patients had both prerenal and renal factors; 56 (7.2%), both prerenal and postrenal factors; 35 (4.5%), both renal and postrenal factors; and 25 (3.2%), all three factors.

**Table 2:** The etiology of AKI in regions

	**Marmara-1**	**Marmara-2**	**Central Anatolia**	**Southern Anatolia**	**Eastern Anatolia**	**Total**	***P***
	n (%)	n (%)	n (%)	n (%)	n (%)	n (%)	
**Prerenal**	164 (65.3)	99 (93.4)	53 (63.9)	170 (74.2)	64 (59.8)	550 (70.9)	***p* < 0.001**
Dehydration	104 (41.4)	78 (73.6)	42 (50.6)	83 (36.2)	33 (30.8)	340 (43.8)	
Heart failure	32 (12.7)	41 (38.7)	8 (9.6)	52 (22.7)	21 (19.6)	154 (19.8)	
Burn	1 (0.4)	0 (0.0)	0 (0.0)	0 (0.0)	0 (0.0)	1 (0.1)	
Gastrointestinal loss	31 (12.4)	17 (16.0)	23 (27.7)	37 (16.2)	13 (12.1)	121 (15.6)	
Liver cirrhosis and ascites	8 (3.2)	2 (1.9)	1 (1.2)	4 (1.7)	2 (1.9)	17 (2.2)	
Sepsis	39 (15.5)	6 (5.7)	10 (12.0)	76 (33.2)	17 (15.9)	148 (19.1)	
**Renal**	112 (44.6)	74 (69.8)	44 (53.0)	165 (72.1)	62 (57.9)	457 (58.9)	***p* < 0.001**
Ischemia	19 (7.6)	41 (38.7)	9 (10.8)	29 (12.7)	21 (19.6)	119 (15.3)	
Nephrotoxic agent	66 (26.3)	28 (26.4)	25 (30.1)	102 (44.5)	32 (29.9)	253 (32.6)	
Aminoglycoside﻿	2 (0.8)	3 (2.8)	1 (1.2)	2 (0.9)	2 (1.9)	10 (1.3)	
Colistin	1 (0.4)	2 (1.9)	0 (0.0)	2 (0.9)	3 (2.8)	8 (1.0)	
Radiocontrast material	28 (11.2)	3 (2.8)	3 (3.6)	19 (8.3)	9 (8.4)	62 (8.0)	
NSAIDs	15 (6.0)	12 (11.3)	5 (6.0)	48 (21.0)	12 (11.2)	92 (11.9)	
RAS blocker	8 (3.2)	1 (0.9)	12 (14.5)	52 (22.7)	6 (5.6)	79 (10.2)	
Chemotherapeutic drugs	7 (2.8)	7 (6.6)	3 (3.6)	0 (0.0)	1 (0.9)	18 (2.3)	
Others	12 (4.8)	9 (8.5)	14 (16.9)	35 (15.3)	6 (5.6)	76 (9.8)	
Glomerulonephritis	12 (4.8)	8 (7.5)	4 (4.8)	15 (6.6)	0 (0.0)	39 (5.0)	
Vasculitis	5 (2.0)	0 (0.0)	7 (8.4)	5 (2.2)	0 (0.0)	17 (2.2)	
Diabetes mellitus	21 (8.4)	14 (13.2)	1 (1.2)	63 (27.5)	5 (4.7)	104 (13.4)	
Tubulointerstitial nephritis	9 (3.6)	6 (5.7)	8 (9.6)	5 (2.2)	7 (6.5)	35 (4.5)	
**Postrenal**	41 (16.3)	16 (15.1)	15 (18.1)	19 (8.3)	14 (13.1)	105 (13.5)	*p* = 0.068
Stone disease	12 (4.8)	2 (1.9)	4 (4.8)	1 (0.4)	4 (3.7)	23 (3.0)	
Malignancy	14 (5.6)	7 (6.6)	6 (7.2)	8 (3.5)	6 (5.6)	41 (5.3)	
Vesicoureteral reflux	1 (0.4)	0 (0.0)	1 (1.2)	1 (0.4)	1 (0.9)	4 (0.5)	
Prostatic disease	16 (6.4)	9 (8.5)	4 (4.8)	9 (3.9)	3 (2.8)	41 (5.3)	
Retroperitoneal fibrosis	0 (0.0)	0 (0.0)	1 (1.2)	0 (0.0)	1 (0.9)	2 (0.3)	

*RAS* Renin-angiotensin-aldosteron system blocker

The prerenal factors were more prevalent in the Marmara-2 and Southern Anatolian regions. Dehydration and heart failure were more frequent in the Marmara-2 region, while sepsis was more prevalent in the Southern Anatolian region. The renal factors were also more frequent in the Marmara-2 and Southern Anatolian regions. The ischemic factors were more frequently reported in the Marmara-2 region, while exposure to nephrotoxic agents, mostly radiocontrast material and RAS blockers, was more prevalent in the Southern Anatolian region than in the other regions. The prevalence of the postrenal factors was similar in all regions.

The causes of hospitalization and comorbidities are presented in Table 3. The most frequent cause of hospitalization was genitourinary diseases, followed by infections and cardiovascular diseases. The most frequent comorbidities were hypertension and DM. 33.6% of the patients were found to have CKD.

**Table 3:** Causes of hospitalization and comorbidities

	**Marmara-1**	**Marmara-2**	**Central Anatolia**	**Southern Anatolia**	**Eastern Anatolia**	**Total**	***P***
	n (%)	n (%)	n (%)	n (%)	n (%)	n (%)	
**Cause of hospitalization**							
Respiratory diseases	49 (19.5)	40 (37.7)	7 (8.4)	54 (23.6)	24 (22.4)	174 (22.4)	** < 0.001**
Cardiovascular diseases	34 (13.5)	56 (52.8)	10 (12.0)	66 (28.8)	26 (24.3)	192 (24.7)	**< 0.001**
Cerebrovascular diseases	9 (3.5)	5 (4.7)	1 (1.2)	12 (5.2)	10 (9.3)	37 (4.8)	0.086
Hematological diseases	8 (3.1)	11 (10.4)	8 (9.7)	20 (8.7)	2 (1.9)	49 (6.3)	**0.006**
Immunosuppressive state	5 (1.9)	6 (5.7)	5 (6.0)	38 (16.6)	0 (0)	54 (7.0)	**< 0.001**
Gastroenterological diseases	48 (19.1)	22 (20.7)	27 (32.5)	30 (13.1)	11 (10.3)	138 (17.8)	**< 0.001**
Hepatological diseases	5 (2.0)	0	0 (0)	7 (3.1)	0 (0)	12 (1.5)	0.078
Genitourinary system diseases	94 (37.4)	57 (53.8)	62 (74.7)	122 (53.3)	55 (51.4)	390 (50.3)	**< 0.001**
Infectious diseases	50 (19.9)	23 (21.7)	15 (18.1)	113 (49.3)	35 (32.7)	236 (30.4)	**< 0.001**
Cardiovascular surgery	3 (1.2)	8 (7.5)	1 (1.2)	7 (3.1)	1 (0.9)	20 (2.6)	**0.006**
Major non-cardiac surgery	17 (6.8)	10 (9.4)	5 (6.02)	13 (5.7)	2 (1.9)	47 (6.1)	0.222
Minor non-cardiac surgery	5 (2.0)	1 (0.9)	0 (0)	4 (1.7)	1 (0.9)	11 (1.4)	0.678
**Comorbidities**						702 (90.5)	
Diabetes Mellitus	91 (36.2)	29 (27.3)	19 (22.9)	93 (40.6)	34 (31.8)	266 (34.3)	**0.019**
Hypertension	132 (52.6)	68 (64.1)	41 (49.4)	127 (55.4)	66 (61.7)	434 (55.9)	0.139
Chronic kidney disease	83 (33.1)	38 (35.8)	14 (16.9)	91 (39.7)	35 (32.7)	261 (33.6)	**0.006**
Ischemic heart disease	54 (21.5)	46 (43.4)	12 (14.4)	84 (36.7)	29 (27.1)	225 (29.0)	**< 0.001**
Chronic obstructive pulmonary disease	20 (7.9)	4 (3.8)	3 (3.6)	17 (7.4)	5 (4.7)	49 (6.3)	0.366
Liver cirrhosis	9 (3.6)	1 (0.9)	0 (0)	7 (3.1)	1 (0.9)	18 (2.3)	0.193
Malignancy	51 (20.3)	20 (18.9)	15 (18.1)	35 (15.3)	12 (11.2)	133 (17.1)	0.262
Heart failure	27 (10.7)	25 (23.6)	11 (13.2)	52 (22.7)	16 (14.9)	131 (16.9)	**0.002**
Others	66 (26.3)	17 (16.0)	13 (15.7)	92 (40.2)	21 (19.6)	209 (26.9)	**< 0.001**

Chi square test

The number of patients with stage 1, 2, and 3 AKI according to the KDIGO classification was 185 (23.9), 259 (33.4%), and 331 (42.7%), respectively. The mean creatinine levels of the patients with stage 1, 2, and 3 AKI were 2.46 ± 1.26, 3.17 ± 1.40, and 5.89 ± 3.60 mg/dL, respectively. The corresponding eGFRs were 74.0 ± 55.4, 48.3 ± 34.8, and 47.8 ± 41.0 mL/min/1.73 m^2^, respectively. Regarding the diagnostic criteria for SIRS, a body temperature more than 38 °C or less than 36 °C was recorded in 135 (17.4%) patients; heart rate above 90 beats/min in 190 (24.5%) patients; respiratory rate exceeding 20 breaths/min or PaCO_2_ level above 32 mmHg in 133 (17.1%) patients; and white blood cell count above 12000/mm^3^ or below 4000/mm^3^ or ratio of band forms more than 10% in 214 (27.6%) patients. A total of 491 (63.3%) patients showed no diagnostic criteria for SIRS; meanwhile, 87 (11.2%) patients showed one criterion; 63 (8.1%), two criteria; 81 (10.4%), three criteria; and 54, (7.0%), four criteria. Sepsis was recorded in 179 (23.1%) patients.

During follow-up, 254 (33.6%) patients needed KRT. Specifically, 229 patients needed IHD; 8 patients, IHD and CKRT; 3 patients, IHD and PD; and 1 patient, PD. The frequency of use of KRT significantly differed among the regions (*p* < 0.001) (Table 4). KRT was most commonly needed among the patients from the Central Anatolia region and least needed among those from the Marmara-2 region.

**Table 4:** The frequencies of use of kidney replacement therapies in the regions

KRT	Marmara-1 n (%)	Marmara-2 n (%)	Central Anatolia n (%)	Southern Anatolia n (%)	Eastern Anatolia n (%)
None	155 (64.3)	79 (77.5)	40 (48.8)	155 (68.6)	72 (69.2)
IHD	71 (29.5)	18 (17.6)	42 (51.2)	68 (30.1)	30 (28.8)
PD	0 (0)	1 (0.9)	0 (0)	0 (0)	0 (0)
CKRT	6 (2.5)	0 (0)	0 (0)	1 (0.4)	1 (1.0)
HD + PD	0 (0)	2 (2.0)	0 (0)	1 (0.4)	0 (0)
HD + CRRT	9 (3.7)	2 (2.0)	0(0)	1 (0.4)	1 (1.0)

*KRT* Kidney replacement therapy, *IHD* Intermittent hemodialysis, *PD* Peritoneal dialysis, *CKRT* Continuous kidney replacement therapies

**Fig. 1:**
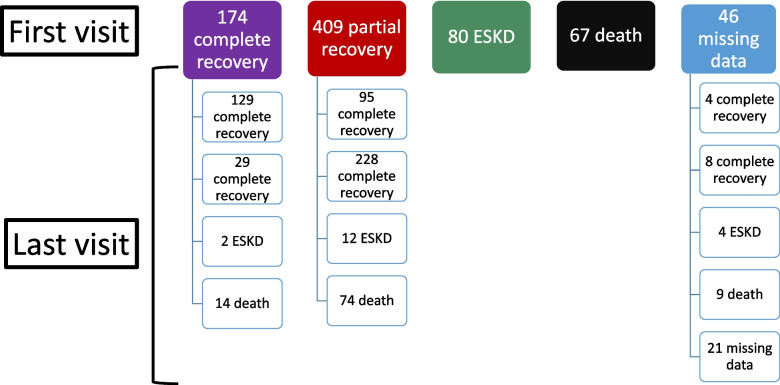
Outcome of patients during first and last controls

At the first follow-up visit of the 776 patients included, 174 recovered completely; 409 partially recovered; 80 received KRT; and 67 died. At the 6^th^ month of diagnosis, 229 (29.5%) recovered completely; 265 (34.1%) partially recovered; 74 (9.5%) developed ESKD; and 187 (24.1%) died. Mortality was related and unrelated to AKI in 124 (66.3%) and 63 (33.7%) patients, respectively. The outcome data could not be reached for 21 patients. The outcomes of the patients at the first and last visits are presented schematically in Fig. 1.

The comparison of the patients grouped according to the outcome at the last visit is presented in Table 5. The male and female patients had similar outcomes. In the comparison of the different geographical groups, the prevalence of complete recovery, partial recovery, and ESKD was the highest in the Southern Anatolian (54.9%), Marmara-2 (35.6%), and Central Anatolian regions (17.1%), respectively. The recovery rate was lower, and the mortality rate was higher in the patients who were admitted to the ICU than in the other patients. The proportion of patients who died or became dialysis-dependent was higher in the patients with stage 3 AKI than in the patients with stage 1 or 2 AKI. The recovery rate decreased, and the risk of mortality and ESKD increased, as the number of the diagnostic criteria for SIRS increased. The outcome was worse in the patients with sepsis, hypertension, CKD, heart failure, and malignancy than in the other patients.

**Table 5:** The comparison of the patients grouped according to the outcome at the last visit

		**Complete recovery**		**Partial recovery**			**Death**		**ESKD**		
		N	%	N	%	N		%	n	%	*P*
**Sex**	Female	111	32.2	128	37.1	78		22.6	28	8.1	0.215
	Male	118	28.8	137	33.4	109		26.6	46	11.2	
**Regions**	Marmara-1	66	27.4	81	33.6	60		24.9	34	14.1	** < 0.001**
	Marmara-2	18	17.6	56	54.9	22		21.6	6	5.9	
	Central Anatolia	32	39.0	26	31.7	10		12.2	14	17.1	
	Southern Anatolia	91	40.3	65	28.8	58		25.7	12	5.3	
	Eastern Anatolia	22	21.2	37	35.6	37		35.6	8	7.7	
**The place of diagnosis**	Inpatient clinic	113	32.3	127	36.3	75		21.4	35	10.0	** < 0.001**
	Intensive care unit	22	17.5	24	19.0	77		61.1	3	2.4	
	Emergency clinic	83	33.5	99	39.9	34		13.7	32	12.9	
**Etiology**	Prerenal	65	27.7	76	32.3	76		32.3	18	7.7	**0.035**
	Renal	46	27.2	71	42.0	27		16.0	25	14.8	
	Postrenal	14	35.9	11	28.2	11		28.2	3	7.7	
	Prerenal + postrenal	10	34.5	12	41.4	5		17.2	2	6.9	
	Prerenal + renal	82	33.6	79	32.4	58		23.8	25	10.2	
	Renal + postrenal	4	40.0	5	50.0	1		10.0	0	0.0	
	All	8	33.3	8	33.3	8		33.3	0	0.0	
**Staging**	Stage-1	52	29.4	87	49.2	30		16.9	8	4.5	** < 0.001**
	Stage-2	73	29.0	103	40.9	55		21.8	21	8.3	
	Stage-3	104	32.0	75	23.1	101		31.1	45	13.8	
**SIRS criteria number**	0	151	31.5	184	38.3	95		19.8	50	10.4	** < 0.001**
	1	34	40.5	33	39.3	10		11.9	7	8.3	
	2	22	36.1	17	27.9	13		21.3	9	14.8	
	3	17	22.4	21	27.6	34		44.7	4	5.3	
	4	5	9.3	10	18.5	35		64.8	4	7.4	
**Sepsis**	Yes	40	23.3	36	20.9	84		48.8	12	7.0	** < 0.001**
	No	189	32.4	229	39.3	103		17.7	62	10.6	
**DM**	Yes	77	29.7	88	34.0	67		25.9	27	10.4	0.911
	No	152	30.6	177	35.7	120		24.2	47	9.5	
**HT**	Yes	107	25.2	169	39.8	105		24.7	44	10.4	**0.002**
	No	122	37.0	96	29.1	82		24.8	30	9.1	
**CKD**	Yes	56	22.0	109	42.7	52		20.4	38	14.9	** < 0.001**
	No	173	34.6	156	31.2	135		27.0	36	7.2	
**Ischemic cardiac/vascular disease**	Yes	58	26.5	81	37.0	63		28.8	17	7.8	0.163
	No	171	31.9	184	34.3	124		23.1	57	10.6	
**Chronic obstructive pulmonary disease**	Yes	12	26.1	15	32.6	15		32.6	4	8.7	0.647
	No	217	30.6	250	35.3	172		24.3	70	9.9	
**Cirrhosis**	Yes	4	23.5	3	17.6	9		52.9	1	5.9	0.077
	No	225	30.5	262	35.5	178		24.1	73	9.9	
**Malignancy**	Yes	30	23.4	36	28.1	53		41.4	9	7.0	** < 0.001**
	No	199	31.7	229	36.5	134		21.4	65	10.4	
**Heart Failure**	Yes	35	27.1	41	31.8	46		35.7	7	5.4	**0.009**
	No	194	31.0	224	35.8	141		22.5	67	10.7	

Chi square test

**Fig. 2:**
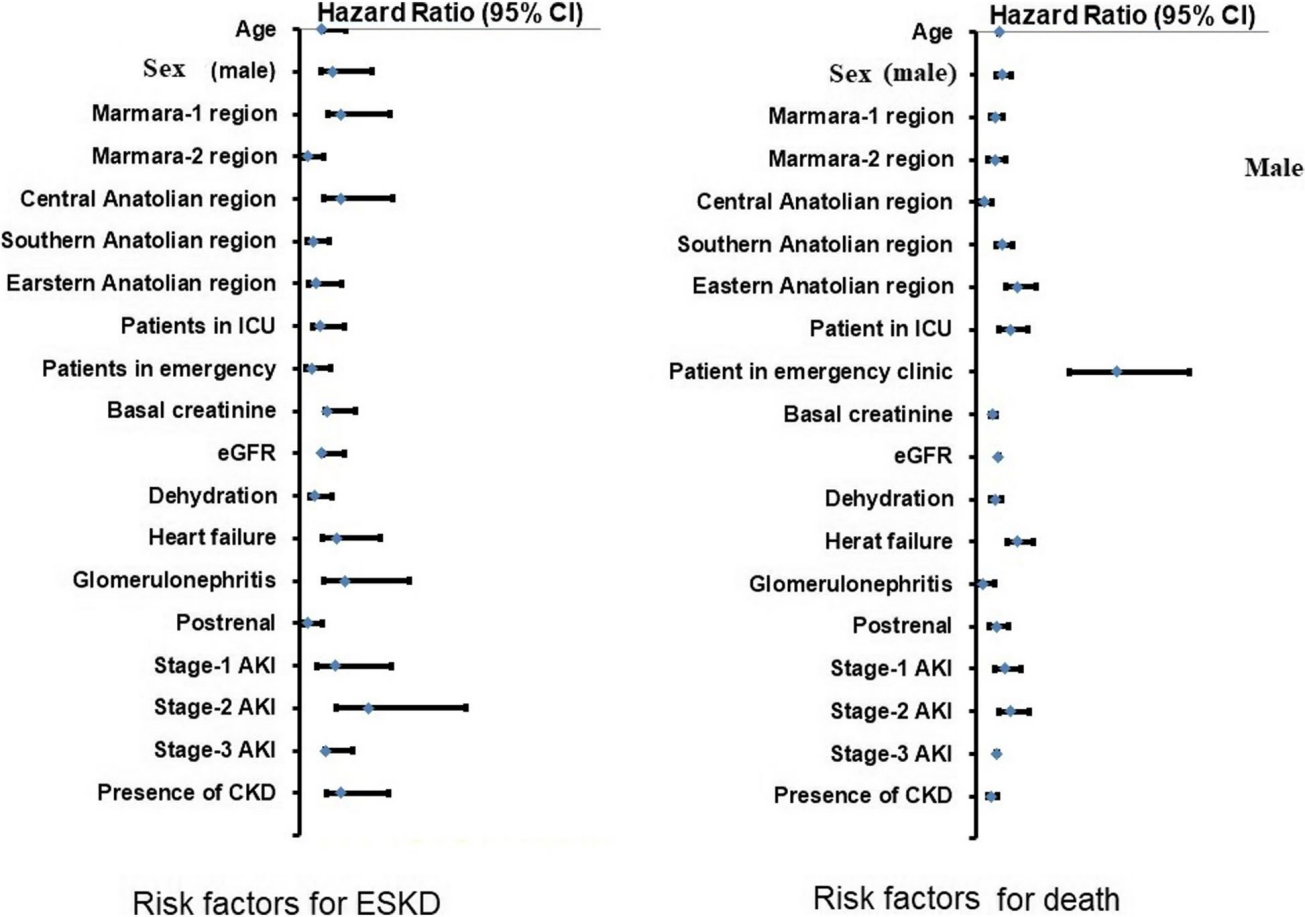
Factors for the risk of ESKD and death determined by Cox regression analysis

The risk of ESKD was higher in the patients from the Central Anatolian region, those with stage 3 AKI, and those with underlying CKD (Table 6) than in the other patients. A Cox regression analysis was performed to determine the risk factors for of ESKD (Fig. 2). Being in the Marmara-1 (HR: 1.8, 95% CI: 1.213–2.684; *p* = 0.004) and Central Anatolian regions (HR: 1.785; 95% CI: 1.070–2.979; *p* = 0.027), having an elevated basal creatinine level (HR: 1.216; 95% CI: 1.130–1.309; *p* < 0.001), having heart failure (HR: 1.590; 95% CI: 1.013–2.495; *p* = 0.044), having glomerulonephritis (HR: 1.956; 95% CI: 1.041–3.675; *p* = 0.037), having stage 2 AKI (HR: 2.968; 95% CI: 1.560–5.649; *p* = 0.001), and having CKD (HR: 1.776; 95% CI: 1.190–2.650; *p* = 0.005) were found to increase the risk of ESKD; meanwhile, being in the Marmara-2 (HR: 0.390; 95% CI: 0.170–0.891; *p* = 0.025) and Southern Anatolian regions (HR: 0.587; 95% CI: 0.359–0.959; *p* = 0.034), having dehydration (HR: 0.649; 95% CI: 0.428–0.985; *p* = 0.042) or postrenal factors (HR: 0.361; 95% CI: 0.158–0.825; *p* = 0.016) as the etiology of AKI, and having stage 1 AKI were associated with a decreased risk of ESKD.

**Table 6:** Factors associated with the risk of end stage kidney disease

		N	%	*P*
Sex	Female	38	11.0	0.117
	Male	61	14.9	
Regions	Marmara-1	45	18.7	< 0.001
	Marmara-2	6	5.9	
	Central Anatolia	18	22.0	
	Southern Anatolia	20	8.8	
	Eastern Anatolia	10	9.6	
The place of AKI diagnosis	Inpatient clinic	49	14.0	0.087
	Intensive care unit	9	7.1	
	Emergency clinic	37	14.9	
Etiology	Prerenal	32	13.6	0.205
	Renal	29	17.2	
	Postrenal	4	10.3	
	Perenal + postrenal	2	6.9	
	Prerenal + renal	31	12.7	
	Renal + postrenal	0	0.0	
	All	0	0.0	
Staging	Stage 1	11	6.2	< 0.001
	Stage 2	25	9.9	
	Stage 3	63	19.4	
Number of SIRS criteria	0	63	13.1	0.367
	1	7	8.3	
	2	12	19.7	
	3	11	14.5	
	4	6	11.1	
Sepsis	Yes	23	13.4	0.909
	No	76	13.0	
Diabetes Mellitus	Yes	38	14.7	0.359
	No	61	12.3	
Hypertension	Yes	58	13.6	0.621
	No	41	12.4	
CKD	Yes	45	17.6	0.008
	No	54	10.8	
Ischemic cardiovascular disease	Yes	22	10.0	0.111
	No	77	14.4	
Chronic obstructive pulmonary disease	Yes	7	15.2	0.663
	No	92	13.0	
Cirrhosis	Yes	3	17.6	0.479
	No	96	13.0	
Malignancy	Yes	19	14.8	0.524
	No	80	12.8	
Heart failure	Yes	12	9.3	0.159
	No	87	13.9	

Chi square test

The comparison of the subgroups according to the mortality rate is presented in Table 7. The mortality rate was higher in the patients from the Eastern Anatolian region; those admitted to the ICU; those with prerenal, renal, and postrenal factors together; those with stage 3 AKI, sepsis, increased number of met diagnostic criteria for SIRS, liver cirrhosis, heart failure, and malignancy; and those without CKD than in the other patients. The mortality analysis was repeated within the group with prerenal etiologies. The patients with AKI due to hypovolemia were compared with those with heart failure and liver cirrhosis. The 6-month survival rates were not significantly different (77.7% and 73.0%, respectively; *p* = 0.316). The Cox regression analysis (Fig. 2) revealed that advanced age (HR: 1.027; 95% CI: 1.017–1.037; *p* < 0.001), number of days between hospitalization and AKI diagnosis (HR: 1.023; 95% CI: 1.013–1.032; *p* < 0.001), admission to the ICU (HR: 1.554; 95% CI: 1.035–2.333; p = 0.034), diagnosis at the emergency clinic (HR: 6.285; 95% CI: 4.174–9.463; *p* < 0.001), stage 3 AKI (HR: 1.563; 95% CI: 1.037–2.355; *p* = 0.033), body temperature above 38 °C or below 36 °C (HR: 2.528; 95% CI: 1.889–3.382; *p* < 0.001), heart rate above 90 beats/min (HR: 2.742; 95% CI: 2.028–3.707; *p* < 0.001), tachypnea (HR: 1.875; 95% CI: 1.399–2.513; *p* < 0.001), leukocytosis or leukopenia (HR: 1.365; 95% CI: 1.247–1.494; *p* < 0.001), diagnostic criteria for SIRS (HR: 3.142; 95% CI: 2.353–4.195; *p* < 0.001), sepsis (HR: 3.142; 95% CI: 2.353–4.195; *p* < 0.001), respiratory disorders (HR: 1.986; 95% CI: 1.470–2.683; *p* < 0.001), cerebrovascular events (HR: 1.976; 95% CI: 1.145–3.409; *p* = 0.014), hepatological diseases (HR: 2.360; 95% CI: 1.108–5.028; *p* = 0.016), infectious diseases (HR: 1.470; 95% CI: 1.093–1.976; *p* = 0.011), cardiovascular surgery (HR: 2.190; 95% CI: 1.120–4.281; *p* = 0.022) as the cause of hospitalization, presence of liver cirrhosis (HR: 2.338; 95% CI: 1.195–4.574; *p* = 0.013), malignancy (HR: 1.811; 95% CI: 1.317–2.490; *p* < 0.001), and heart failure (HR: 1.791; 95% CI: 1.282–2.500; *p* = 0.001) increased the risk of mortality. Meanwhile, glomerulonephritis (HR: 0.307; 95% CI: 0.114–0.827; *p* = 0.02) or genitourinary diseases as the etiology of AKI (HR: 0.583; 95% CI: 0.432–0.785; *p* < 0.001) and underlying CKD (HR: 0.698; 95% CI: 0.507–0.963; *p* = 0.028) were found to decrease the risk of mortality. The multivariate analysis was performed including parameters related to the mortality rate in the univariate analysis as well as the demographical parameters. Advanced age (HR: 1.025; 95% CI: 1.013–1.038; *p* < 0.001), increased interval between the admission and diagnosis of AKI (HR: 1.017; 95% CI: 1.006–1.029; *p* = 0.002), heart failure (HR: 1.773; 95% CI: 1.226–2.565; *p* = 0.002), liver cirrhosis (HR: 2.504; 95% CI: 1.235–5.077; *p* = 0.011), sepsis (HR: 2.537; 95% CI: 1.664–3.868; *p* < 0.001), and malignancy (HR: 1.982; 95% CI: 1.376–2.856; *p* < 0.001) were associated with an increased risk of mortality. Being in the Central Anatolian region (HR: 0.476; 95% CI: 0.239–0.947; *p* = 0.035), having gastrointestinal losses as the etiology of AKI (HR: 0.511; 95% CI: 0.292–0.895; *p* = 0.019), having genitourinary diseases as the cause of hospitalization (HR: 0.644; 95% CI: 0.450–0.922; *p* = 0.016), and having CKD as the comorbidity (HR: 0.636; 95% CI: 0.434–0.931; *p* = 0.020) were associated with better survival. The effect of the stage of CKD was analyzed among subgroups including patients with stage 1–2, 3, 4, and 5 CKD. The 6-month survival rates of these subgroups were 70.2%, 70.4%, 81.4%, and 81.1%, respectively. The mortality rate was higher in the patients with stage 1–2 CKD than in those with stage 4 (*p* = 0.022) and 5 CKD (*p* = 0.006). Further, the mortality rate was higher in the patients with stage 3 CKD than in those with stage 5 CKD (*p* = 0.038).

**Table 7:** The comparison of the subgroups according to the mortality rate

		Death		P*
		N	%	
Sex	Female	78	22.6	0.207
	Male	109	26.6	
Regions	Marmara-1	60	24.9	0.007
	Marmara-2	22	21.6	
	Central Anatolia	10	12.2	
	Southern Anatolia	58	25.7	
	Eastern Anatolia	37	35.6	
The place of AKI diagnosis	Inpatient clinic	75	21.4	< 0.001
	Intensive care unit	77	61.1	
	Emergency clinic	34	13.7	
Etiology	Prerenal	76	32.3	0.007
	Renal	27	16.0	
	Postrenal	11	28.2	
	Perenal + postrenal	5	17.2	
	Prerenal + renal	58	23.8	
	Renal + postrenal	1	10.0	
	All	8	33.3	
Staging	Stage 1	30	16.9	0.001
	Stage 2	55	21.8	
	Stage 3	101	31.1	
Number of SIRS criteria	0	95	19.8	< 0.001
	1	10	11.9	
	2	13	21.3	
	3	34	44.7	
	4	35	64.8	
Sepsis	Yes	84	48.8	< 0.001
	No	103	17.7	
Diabetes Mellitus	Yes	67	25.9	0.613
	No	120	24.2	
Hypertension	Yes	105	24.7	0.954
	No	82	24.8	
CKD	Yes	52	20.4	0.047
	No	135	27.0	
Ischemic cardiovascular disease	Yes	63	28.8	0.104
	No	124	23.1	
COPD	Yes	15	32.6	0.204
	No	172	24.3	
Cirrhosis	Yes	9	52.9	0.018
	No	178	24.1	
Malignancy	Yes	53	41.4	< 0.001
	No	134	21.4	
Heart failure	Yes	46	35.7	0.002
	No	141	22.5	

Chi square test

**Fig. 3:**
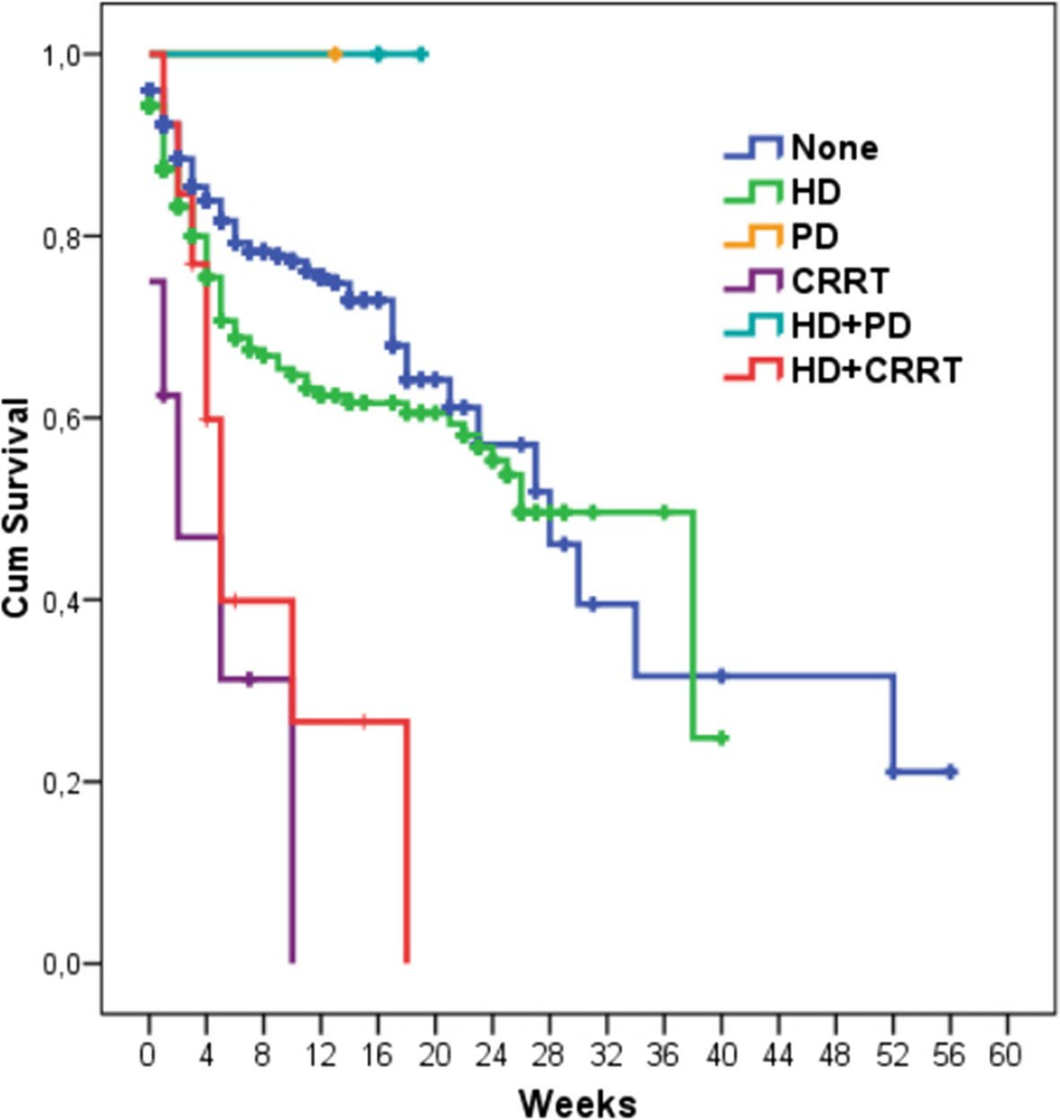
Mortality analysis of patients who needed KRT and those who did not

**Fig. 4:**
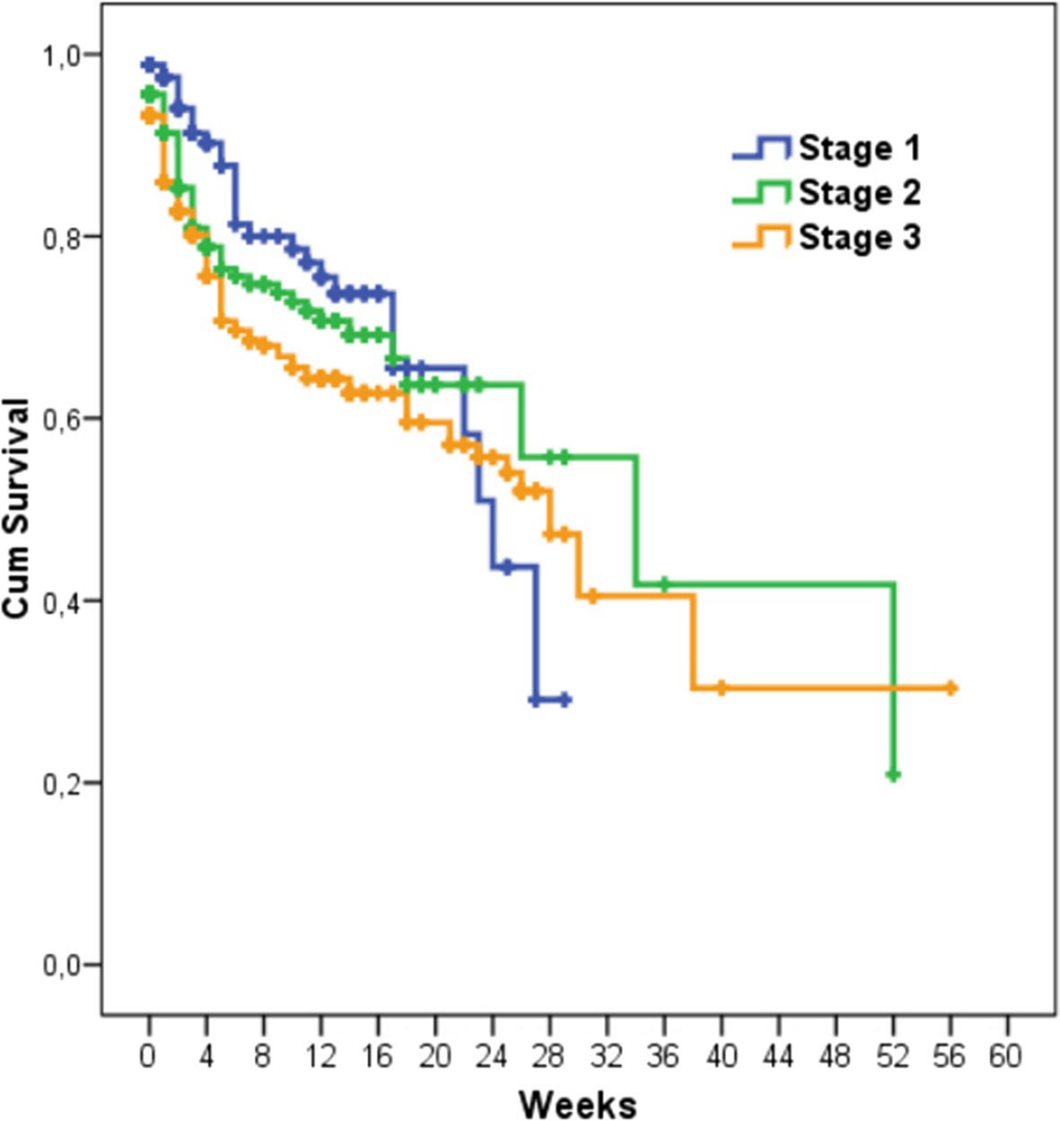
Mortality analysis of patients with different stages of acute kidney injury

The mortality analysis results of the patients who needed KRT and those who did not are presented in Fig. 3. The 6-month cumulative survival rate was higher in the patients who did not need KRT than in those who needed IHD (79.3% vs. 68.8%; *p* < 0.001), CKRT (79.3% vs. 31.3%; *p* < 0.001), and IHD plus CKRT (79.3% vs. 39.9%; *p* < 0.001). The 6-month survival rates of the patients with stage 1, 2, and 3 AKI were 43.7%, 63.7%, and 55.7%, respectively (Fig. 4). The difference between the patients with stage 1 and 2 AKI was significant (*p* = 0.030).

## Discussion

AKI is defined as a clinical situation with heterogeneous etiology, clinical presentation, and both renal and patient survival. Its incidence is highly dependent on the study population. For example, Srisawat et al. [9] studied critically ill patients admitted to the ICU and reported that 32% of the study population (*n* = 15132) developed AKI. Another study reported an incidence of 12.7% [10]. In our study, we decided to evaluate the data of patients with AKI diagnosed by nephrology specialists at emergency clinics, inpatient wards, or ICUs.

Compared with Europe and Asia, Turkey is a crowded country with a population of 81623817 in its 814578 km^2^ surface area according to the records of the Turkish Statistical Institute [11]. It is divided into seven different geographical areas, each with different characteristics related to climate, flora, and residents. The majority of the population resides in the Western regions, followed by the Mediterranean region, an eastern part of Turkey. There are enough nationwide technical opportunities/personnel for the care of patients with AKI, such as nephrologists, educated nurses and technicians, modern hospitals and ICUs, and dialysis facilities, including those for all types of KRT. Moreover, the social security system covers renal care of patients both in university and government hospitals nationwide. Thus, there is no obstacle in caring for patients with AKI regarding coverage by the social security system.

A sufficiently large number of cases with AKI (*n* = 776) were included in our study to evaluate the etiologic factors, geographical differences, and outcomes. Unfortunately, we could not conclude on the incidence of AKI, since we did not have data on the number of patients admitted to the related centers during the study period. Further, not all hospitals and nephrologists provided data to the study. Therefore, we could not evaluate the proportion of patients with AKI within the outpatient and inpatient populations. Nevertheless, we were able to evaluate the clinical courses, treatment options, and differences between the geographical regions.

Many disease conditions are known to contribute to the development of AKI. The incidence of AKI may differ according to the features of the studied population and approach of the physician. The nephrological approach in patients at high risks can be lifesaving in this field. Training physicians on how to recognize and evaluate patients at high risks to protect them from developing AKI is important. To clarify the different features among countries, the International Society of Nephrology (ISN) designed the Snapshot Study [12], which was a multinational study including 3664 patients from different countries with varying degrees of income. Hypotension or shock was the most common etiological factor in high-income countries; meanwhile, dehydration was the most common factor in lower-income countries, wherein sepsis and AKI related to pregnancy were more frequent [12]. In a study conducted in Malawi, community-acquired AKI was detected in 12.7% of admissions within a pre-specified period of 3 months. A significant number of patients (43.8%) were seropositive for HIV. The most frequent causes of AKI were reported to be HIV-related sepsis and hypovolemia mostly due to gastroenteritis. Toxins, urinary obstruction, and primary renal parenchymal diseases were less prevalent [10]. Investigators from Karachi studied hospital-acquired AKI and found that the most common causes of AKI were sepsis, gastroenteritis, and surgical and obstetric complications [3]. Another study conducted in Singapore analyzed 422 cases of AKI. The most common etiological factors were prerenal etiologies, followed by sepsis and ischemic acute tubular necrosis [13]. Lombardy et al. [14] studied the causes of AKI in 2864 patients. They reported that AKI was more prevalent during winter (RR: 1.16; 95% CI: 1.05–1.29; *p* = 0.003), and a higher AKI risk was found to be associated with lower air temperature and higher humidity [14]. In our study, the most frequent etiology of prerenal AKI was dehydration, heart failure, and sepsis. This prompts the necessity of early evaluation and supportive care of patients with symptoms that may cause dehydration. With advances in the treatment options for ischemic heart disease, survival is prolonged, and an increasing number of cardiorenal syndrome cases are observed. There is a need for education among both primary care physicians and patients regarding the use of diuretics, especially their correct indication and dose, to prevent both hypervolemia and dehydration.

Since the primary function of the kidneys is concentration and excretion of toxic metabolites and drugs, the kidneys are the main organ affected by drug toxicity. It has been reported that nephrotoxicity accounts for 8–60% of all AKI cases depending on the population and definition of AKI [15]. Interestingly, the use of nephrotoxic agents was the most prominent etiology of renal AKI in our population (72.1%). This result was found to be true for all parts of the country. Thus, it is important to alert all physicians regarding nephrotoxicity of medications, especially those practicing in ICUs.

Infectious diseases are commonly associated with AKI. Krairoun et al. [16] studied 1716 patients admitted to emergency clinics with suspected infectious disease. Of these patients, 10.8% had AKI, and 12.4% died; 4.2% of those who died had no AKI [16].

Sepsis is also commonly associated and/or confounded with AKI [17]. Deleterious inflammatory events, hypovolemia, and exposure to nephrotoxic agents are some of the many potential causes of AKI seen in patients with sepsis. Infections were among the frequent etiologies of AKI in our study, as in many previous studies, and the proportion of patients with sepsis was quite high. Timely and adequate treatment of infections may prevent the development of AKI.

One of the known major risk factors for AKI is the presence of CKD. Approximately one-third of the patients included in our study had CKD. This necessitates careful evaluation of these patients.

There was some regional variation in the etiology of AKI regarding prerenal and renal factors. Dehydration and heart failure were more frequent in the Marmara-2 region, while sepsis and dehydration were more prevalent in the Southern Anatolia region. This cannot be explained simply by the several possible related factors, such as the change in the population characteristics, including age and sex, climate differences, and predominant population of patients cared for in clinical settings, such as cardiovascular hospitals or cancer centers. Another factor may be related to the physicians. The characteristics of the patients may change if the physician is responsible for inpatient, outpatient, or emergency clinic consultation. This factor also applies to the differences between the causes of hospitalization and comorbidities of patients according to region.

We concluded that the diversity of the type of KRT among the geographical regions in our study may be related to the individual characteristics of the patients and technical facilities or the differences in the practice of the clinicians providing data to the study.

Data on renal and patient survival vary in the literature. Again, the study population, risk factors, and socioeconomic factors may play a role. In one study, the overall mortality rate of patients with AKI admitted to the ICU was reported to be 27%, which was correlated with the severity of AKI [9]. The mortality rate was higher in lower-income countries than in higher-income countries in the ISN Global Snapshot Study [12]. In this study, 22% of patients needed dialysis, and among them, the mortality rate was higher. Age, concomitant organ dysfunction, sepsis, and oliguria were other factors related to increased mortality rates, while the presence of CKD was associated with decreased mortality rates. The mortality rate of patients with prerenal AKI due to hypovolemia was similar to that of patients with prerenal AKI secondary to heart failure or liver cirrhosis. However, in the study by Evans et al. [10], the mortality rate increased with advancing stage of AKI. Age above 40 years, stage of AKI, and history of nephrotoxin exposure were found to increase the risk of mortality related to AKI.

In a study conducted in Singapore, KRT was needed in 27% of patients, and the in-hospital and 6-month mortality rates were reported to be 20.3% and 9.4%, respectively. Stage 3 AKI was associated with higher mortality rates [13]. Another study from India reported that 220 of 1150 patients admitted to a general ward in a year had AKI. The in-hospital mortality rate of patients with and without AKI was reported to be 19.09% and 1.8%, respectively. Hematological malignancies, the need for inotropic agents, and the serum creatinine level at the time of admission were independent predictors of mortality. Underlying CKD and hospital-acquired AKI were not related to mortality [18]. In our study, the mortality rates at the 1^st^ week and 6^th^ month of diagnosis were 8.6% and 24.1%, respectively. This is an alarming finding, necessitating all clinicians to follow up patients in the long term. An increasing stage of AKI was associated with both patient and renal survival, which is common in most reports in the literature. Using the staging system proposed by the KDIGO may help clinicians determine patients at the highest risk.

Besides mortality, AKI is also associated with worse renal outcomes. In a recent study involving 5548 patients receiving anesthesia for the first time, all stages of AKI had been found to be related to progression to CKD [19]. In our study, ESKD developed in 74 (9.5%) patients. Being in the Marmara-1 and Central Anatolian regions and having elevated basal creatinine levels, heart failure, glomerulonephritis, stage 2 AKI, and CKD were found to increase the risk of ESKD; conversely, being in the Marmara-2 and Southern Anatolian regions and having dehydration or postrenal factors as the etiology of AKI were associated with a decreased risk of ESKD.

Regional variations may be related to the different risk factors, etiologies, and clinical practices. Dehydration and postrenal factors seem to cause a potentially reversible AKI. CKD is a well-known risk factor for AKI, as shown in our study. Advanced age, admission to the ICU, diagnosis at the emergency clinic, history of cardiovascular surgery, and presence of stage 3 AKI, the diagnostic criteria of SIRS, sepsis, respiratory diseases, cerebrovascular diseases, hepatological diseases, infectious diseases, cirrhosis, malignancy, and heart failure were related to increased mortality rates in our study. Most of these factors are similar to those in the current literature. In a study from Lithuanian University, 575 cases of severe AKI requiring KRT were analyzed [19]. The lowest mortality rate was noted in patients with postrenal AKI and the highest mortality rate in those with renal causes. Older age, systolic blood pressure below 120 mmHg, multiple organ dysfunction, pH level of < 7.3, oliguria, hepatorenal syndrome, cardiac surgery, and sepsis were associated with very high mortality rates. The AKI risk and mortality rate have been reported to be substantially high in patients with liver cirrhosis [20]. This finding is consistent with our results. Glomerulonephritis as the etiology and underlying CKD were associated with decreased mortality rates. The first association may be attributed to the potential reversibility of the disorder with treatment of glomerulonephritis. The mortality rate was lower in the patients with CKD than in those without and was found to decrease with advancing stage of preexisting CKD, when present. The positive effect of preexisting CKD is an interesting finding that may be speculated to be attributed to the less clinical implication of hyperkalemia in patients with CKD. Moreover, there might be an effect of the population included in the study. As we could not include all patients with AKI admitted to the involved centers, bias might have occurred during evaluation. There might have been patients with advanced CKD and multiple comorbidities with worse prognosis.

Herein, the mortality rate was higher in the patients who needed CKRT than in those who did not. This is an expected finding considering the indications for CKRT, instead of conventional hemodialysis, with the most common one being hemodynamic instability.

## Conclusion

The most common etiology of AKI in our study was dehydration. 58.9% of the patients had at least one renal etiology, among which nephrotoxin exposure was the most frequent. At the 6^th^ month of diagnosis, 33.6% of the patients needed KRT; 29.5% completely recovered; 34.1% partially recovered; 9.5% developed ESKD; and 24.1% died. The difference in the etiology and outcome among the geographical regions may be related to the individual characteristics of the patients included or the differences in the practice patterns of physicians providing data to the study. Taken together, physicians managing patients with AKI should be aware of the patient characteristics and outcomes in their country and region to prevent AKI progression and provide more efficient treatment.

## Data Availability

The datasets generated during and/or analysed during the current study are available from the corresponding author on reasonable request.

## Abbreviations

AKI: Acute kidney injury
CI: Confidence interval
CKD: Chronic kidney disease
CKRT: Continuous kidney replacement therapies
DM: Diabetes mellitus
ESKD: End stage kidney disease
eGFR: Estimated glomerular filtration rate
HIV: Human immunodeficiency virus
HR: Hazard ratio
ICU: Intensive care unit
IHD: Intermittent hemodialysis
IQR: Interquartile range
ISN: International Society of Nephrology
KDIGO: Kidney Disease Improving Global Outcomes
KRT: Kidney replacement therapy
NSAID: Non-steroidal anti-inflammatory drugs
PD: Peritoneal dialysis
RAS: Renin-angiotensin-aldosteron system
SIRS: Systemic inflammatory response syndrome
